# Metabolism of Mycotoxins, Intracellular Functions of Vitamin B_12_, and Neurological Manifestations in Patients with Chronic Toxigenic Mold Exposures. A Review

**DOI:** 10.1100/tsw.2004.133

**Published:** 2004-08-26

**Authors:** Ebere C. Anyanwu, Mohammed Morad, Andrew W. Campbell

**Affiliations:** ^1^ Neurosciences Research, Cahers Inc., 8787 Shenandoah Park Drive, Suite 122, Conroe, Houston, 77385 TX, USA; ^2^ Clalit Health Services and Division of Community Health, Department of Family Medicine, Ben Gurion University, Beer-Sheva, Israel; ^3^ Medical Center for Immune and Toxic Disorders, 20510 Oakhurst Drive, Suite 200, Spring TX, USA

**Keywords:** vitamin B_12_, toxigenic molds, mycotoxins, one-carbon metabolism

## Abstract

This paper evaluates the possible reasons for consistent vitamin B_12_ deficiency in chronic toxigenic mold exposures and the synergistic relationships with the possible mycotoxic effects on one-carbon metabolism that lead to the manifestations of clinical neuropathological symptomology. Vitamins are first defined in general and the nutritional sources of vitamin B_12_ are evaluated in particular. Since patients with chronic exposures to toxigenic molds manifest vitamin B_12_ deficiencies, the role of mycotoxins in vitamin B_12_ metabolism is assessed, and since vitamin B_12_ plays important biochemical roles in one-carbon metabolism, the synergistic effects with mycotoxins on humans are reviewed. An outline of the proposed mechanism by which mycotoxins disrupt or interfere with the normal functions of vitamin B_12_ on one-carbon metabolism is proposed. The overall functions of vitamin B_12_ as a source of coenzymes, in intracellular recycling of methionine, in methionine synthase reaction, in the prevention of chromosome breakage, in methylation, and in maintaining a one-carbon metabolic balance are reviewed. Signs, symptoms, and clinical neurological indications of vitamin B_12_ deficiency are also cited. By implication and derivation, it is likely that the interruption of the structure and function of vitamin B_12_ would in turn interfere with the one-carbon metabolism leading to the neurological manifestations. This review is an attempt to formulate a basis for an ongoing research investigation on the subject.

## INTRODUCTION

Among factors that affect human health and development, vitamins are the most essential. Vitamins are natural organic chemical compounds that can be extracted from animal or vegetable sources or chemically synthesized in the laboratory, but required in trace amounts for health, growth, and reproduction[1]. They are, in essence, often functionally coenzymatic and relatively more reactive than other macronutrients, such as proteins, lipids, and carbohydrates. Although deficiency of a single vitamin is relatively uncommon, it can occur as a result of inborn error of metabolism, unusual restriction in dietary intake, or failure to absorb a single vitamin[2]. However, in practice it has been observed in the last 2 years that a majority of patients presenting with chronic exposures to toxigenic molds have persistent vitamin $B_{12}$ deficiency, which had little or nothing to do with dietary insufficiency. These patients eat well and take appropriate nutrient supplements, however, some of their main neurological manifestations are similar to those found in a typical vitamin $B_{12}$ deficiency (see Table 1). The reason for the persistent vitamin $B_{12}$ deficiency is not understood. However, it is proposed that the mycotoxins must have interfered with major metabolic processes that regulate the biosynthesis, reuptake, and utilization of vitamin $B_{12}$.

**TABLE 1**
**Signs, Symptoms, and Clinical Neurological Indications of Vitamin $B_{12}$ Deficiency**

| Signs and Symptoms | Clinical Neurological Indications |
|---|---|
| Headache, fatigue, loss of appetite | Nerve damage and demyelination |
| Pinky-red sore or smooth tongue | Degeneration of PNS leading to paralysis |
| Growth failure in children | Progressive peripheral neuropathy |
| Psychosis, confusion | Spinal degeneration and macrocytic cells |
| Depression, memory loss | Alzheimer's disease, allergies, asthma |
| Anxiety, insomnia | Bipolar, chronic fatigue syndrome |
| | Crohn's disease, multiple sclerosis |
| | Insomnia, sciatic neuritis |
| | Trigeminal neuralgia, osteoarthritis |

The most common of these is the lack of intrinsic factor secretion by the stomach and intestinal malabsorption. Malabsorption may be caused by inflammatory diseases of the small intestine, intestinal stasis with overgrowth of colonies of bacteria or molds. More frequent are complex deficiencies that arise as a result of food fads; as complications of certain diseases, especially those that affect food absorption; as a result of massive losses of blood or from hemodialysis; and with use of certain drugs[1,3]. Excessive use of some vitamins can lead to vitamin imbalance especially in the Western World[1].

In practice, vitamin $B_{12}$ deficiency that is unconnected with dietary insufficiency has been observed in a majority of patients with chronic exposures to toxigenic molds. These patients also showed some degrees of unexplained neurological disorders. It is suspected that the neurological disorders might be due to an interference with one-carbon metabolism leading to $B_{12}$ deficiency. The aim of this review, therefore, is to examine and assess various biochemical reasons that might have impeded on the normal intracellular functions of vitamin $B_{12}$ that lead to such unexplained neurological manifestations.

## METHODS

Vitamins in general were first defined and the nutritional sources and functions of vitamin $B_{12}$ were cited to gain a better understanding of the interactions of one-carbon metabolism and other biological macromolecules and how mycotoxins could interfere with these interactions. Available literature on vitamin $B_{12}$ deficiencies in chronic toxigenic mold exposures was searched and the possible synergistic relationships with mycotoxins in one-carbon metabolism were evaluated. The processes that could lead to the manifestations of biochemical and neuropathological symptomology are reviewed. An outline of the mechanism by which mycotoxins disrupt or interfere with the normal functions of vitamin $B_{12}$ in one-carbon metabolism is proposed. The overall functions of vitamin $B_{12}$ as a source of coenzymes, in intracellular recycling of methionine, in methionine synthase reaction, in the prevention of chromosome breakage, in methylation, and in maintaining a one-carbon metabolic balance are reviewed.

## DEFINITION

Vitamin $B_{12}$ (cobalamin) is a water-soluble vitamin that is stored in the liver and although sensitive to ultraviolet light, vitamin $B_{12}$ is derived from animal products including liver, beef, kidney, chicken, fish (such as salmon, halibut, and tuna), yogurt, milk, Swiss cheese, American processed cheese, and eggs. Vegetarians may derive vitamin $B_{12}$ from cereals, soya milk, and vitamin $B_{12}$–fortified vegetarian burger patties. In humans, the daily vitamin $B_{12}$ requirement is approximately 0.5 µg. However, the recommended daily intake for adults is 2.0 µg, 2.2–2.6 µg during pregnancy and during breast feeding, respectively[3]. Vitamin $B_{12}$ is essential for carbohydrate, fat, and protein metabolism and serves an important role in the formation and regeneration of red blood cells, and in the maintenance of the central nervous system.

### The Role of Mycotoxin in Vitamin $B_{12}$ Deficiency

The problem of regulating mycotoxin biosynthesis, determining the factors that influence the extent of mycotoxin contamination of food, and devising new methods of reducing or eliminating their contamination of food has become an important aspect of clinical and environmental mycology. Fungal metabolism leads to the production of secondary metabolites, the majority of which are formed from a relatively small number of branch points of primary metabolic pathways. Invariably, some of these secondary metabolites are used as antibiotics, while others are very toxic and carcinogenic to humans and animals. There are well over one hundred known mycotoxins and many of them contaminate cereals, grains, and other foodstuffs worldwide. Additionally, several genera of fungi produce toxins in pasture grasses, producing a variety of problems directly in animals grazing in these pastures and indirectly in the farmers.

There are several pieces of evidence to show that mycotoxins affect cellular activities of the brain for which vitamin $B_{12}$ plays a major role. For example, Riley et al.[4] showed that alteration of sphingolipid metabolism was associated with fumonisin-induced animal diseases, including increased apoptotic and oncotic necrosis with carcinogenesis in animal liver and kidney. It is believed that the biochemical consequences of fumonisin disruption of sphingolipid metabolism most likely to alter cell regulation are increased free sphingoid bases and their 1-phosphates, alterations in complex sphingolipids, and decreased ceramide (CER) biosynthesis. Earlier, Enongene et al.[5] demonstrated the disruption of sphingolipid metabolism in small intestines, liver, and kidney of mice dosed subcutaneously with fumonisin B. Vitamin $B_{12}$ possibly interacts with mycotoxins to effect some of the biochemical and neurological changes, such as was discussed by Crews et al.[6]. It is not yet clear whether vitamin $B_{12}$ deficiency precludes fungal infection or vice versa. However, the factors commonly believed to predispose to recurrent chronic atrophic fungal infections included deficiency in whole blood folate, iron, and vitamin B complex[7]. These atrophies occur as a result of metabolic disorders and are due to inactivity of enzymes that are characteristic of vitamin B complex deficiencies[8]. Vitamin $B_{12}$, in particular, has been implicated in the pathogenesis of several fungal infections such as candidaisis, even though much disagreement exists as to the specific roles played by individual vitamins[9]. Nonetheless, there is little doubt that metabolic and nutritional factors either acting locally or via systemic mechanisms could significantly affect the pathogenesis of fungal infection[10].

## Synergy of Mycotoxins, One-Carbon Metabolism, and Vitamin $B_{12}$ Interactions

One-carbon metabolism is a complex set of biochemical reactions that contribute to many central biosynthetic pathways. Although the regulation of this complex set of reactions is difficult to analyze because of the ability of the reactants to interchangeably supplement the pools of one-carbon-derived compounds, several approaches have been adopted to identify the components of the signaling system that regulates the metabolic activity[11]. One-carbon metabolism is an essential process that relies on one-carbon donor molecules such as serine, glycine, and formate. Cells regulate the balance of one-carbon flow between the donors by regulating cytoplasmic hydroxymethyltransferase activity of the donor in a side reaction. The knowledge of one-carbon metabolism is essentially very important because any failure in the metabolic process is a risk factor for cardiovascular disease, stroke and thrombosis, loss of neurocognitive function and Alzheimer's disease, and similar neurological and psychiatric disturbances including depression, dementia, and a demyelinating myelopathy[12,13,14]. To prevent the occurrence of these metabolic disorders, there has to be a mechanism for the one-carbon metabolism to be homeostatically maintained and regulated. The effect of mycotoxins on one-carbon metabolism that lead to vitamin $B_{12}$ depletion or deficiency and the mechanism by which this happens is not clear. It is envisaged that mycotoxins directly and indirectly may interfere with a number of vitamin $B_{12}$ functions and one-carbon metabolism. By implication, as shown in Fig. 1 for example, improper function of the one-carbon metabolism can lead to abnormal fatty livers and increased liver-cell turnover, thereby promoting carcinogenesis as has been found in experimental animals[15].

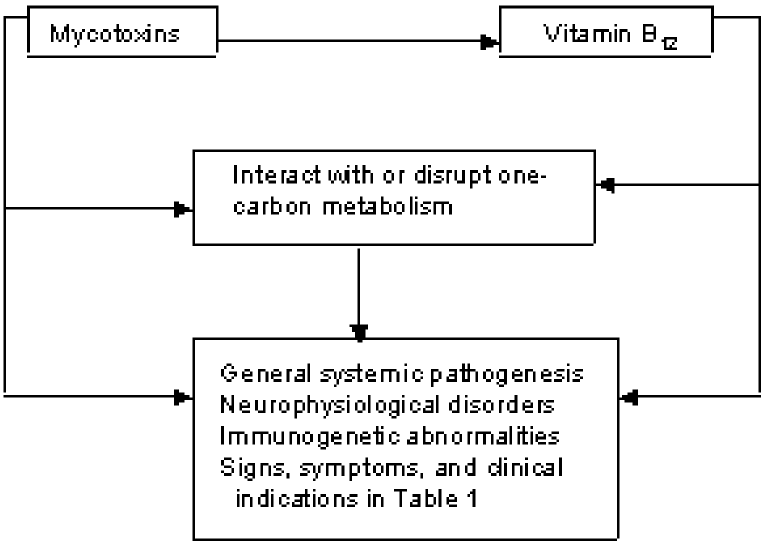

**FIGURE 1.** A proposed outline of the mechanism by which mycotoxins disrupt or interfere with the normal functional effects of vitamin $B_{12}$ on one-carbon metabolism. It is likely that the interruption of the structure and function of vitamin $B_{12}$ would in turn interfere with the one-carbon metabolism leading to the neurological manifestations.

Considering the functional groups within the structure of mycotoxins, it is mechanistically most likely that mycotoxins have the functional properties to deplete the S-adenosylmethionine pools, resulting in DNA hypomethylation, which in turn leads to changes in expression of genes that may have key roles in regulation of growth[15]. Although human diets are unlikely to be as severely methyl deficient as those used in these animal experiments, in some parts of the world (including industrialized nations) intake of diets that are low in methionine and choline and contaminated with mycotoxins, such as aflatoxin, trichothecenes, and *Stachyboytris*, are reported frequently with associated vitamin $B_{12}$ deficiencies. Thus, it seems possible that interactions of food and mycotoxins can induce changes in DNA methylation and aberrant gene expression, hence contributing to cancer development[15].

## Vitamin $B_{12}$ in Maintaining a One-Carbon Metabolic Balance

As stated earlier, one-carbon metabolism comprises a complex set of reactions that contribute to many central biosynthetic pathways. It is therefore a dynamic metabolic system in which one-carbon units are interconverted between the mitochondrial and cytoplasmic compartments, as well as between oxidation states. The importance of this flow has been the subject of extensive biochemical analyses[16] that showed that glycine, serine, and formate could each act to supplement all of the different one-carbon pools[11,17,18,19]. The magnitude of the role of vitamin $B_{12}$ in one-carbon metabolism and the possible effects of mycotoxins on this process can be explained by inferences from Figs. 2 and 3 that described the one-carbon metabolism in yeast[11,17,18,19]. The process relies on at least one of three one-carbon donor molecules: serine, glycine, or formate.

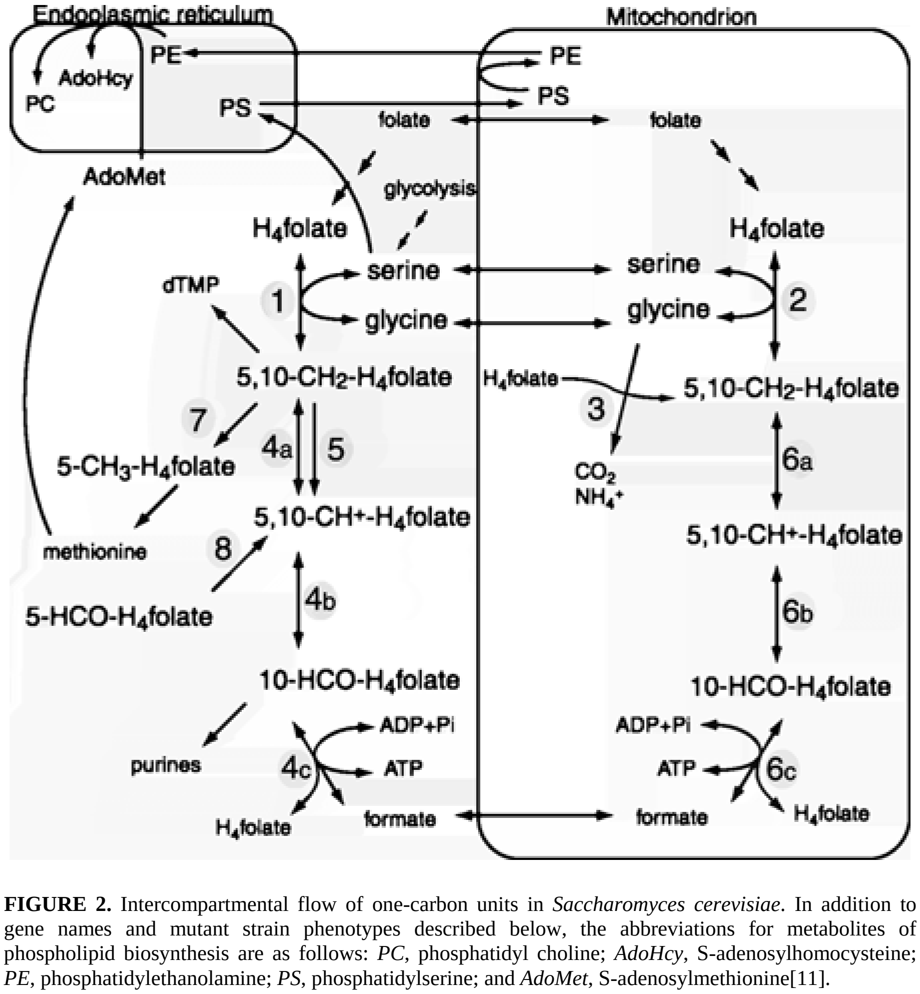

**FIGURE 2.** Intercompartmental flow of one-carbon units in *Saccharomyces cerevisiae*. In addition to gene names and mutant strain phenotypes described below, the abbreviations for metabolites of phospholipid biosynthesis are as follows: *PC*, phosphatidyl choline; *AdoHcy*, S-adenosylhomocysteine; *PE*, phosphatidylethanolamine; *PS*, phosphatidylserine; and *AdoMet*, S-adenosylmethionine[11].

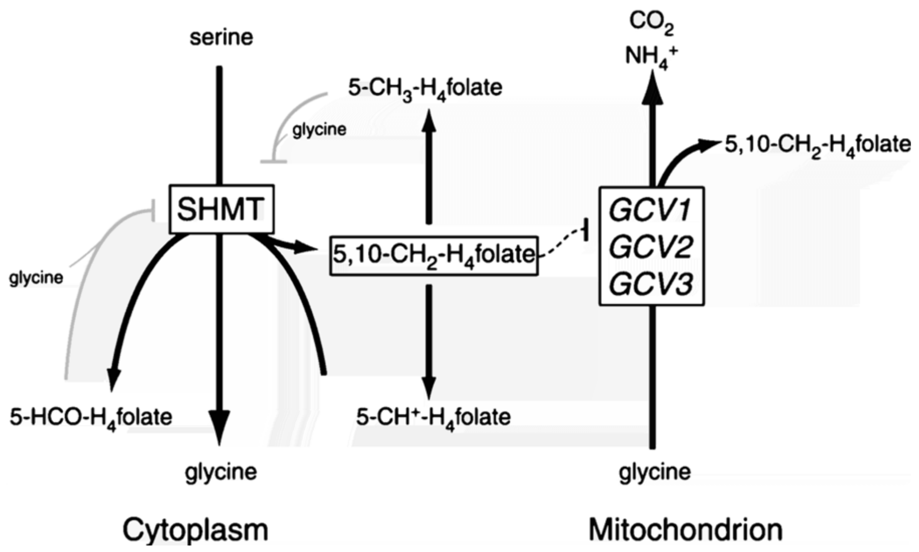

**FIGURE 3.** Model of how cells regulate one-carbon metabolism and gene transcription via the activity of cytoplasmic SHMT. Regulation of SHMT activity by a side reaction dictates cytoplasmic 5,10-$CH_2$-$H_4$ folate levels, which mediate transcription of genes involved in generation of one-carbon units from glycine. This control loop has the effect of shifting the balance of one-carbon metabolism toward the mitochondrial reactions. *Solid black arrows* indicate enzyme-catalyzed reactions; *gray lines* indicate control of enzyme activity; and the *dashed line* indicates regulation of gene expression[11,20,21,22,23].

Cells regulate the balance of one-carbon flow between the donors by regulating cytoplasmic serine hydroxymethyltransferase activity in a side reaction occurring in the presence of excess glycine[11]. This control governs the level of 5,10-methylene tetrahydrofolate (5,10-$CH_2$-$H_4$ folate) in the cytoplasm (see Fig. 3), which has a direct role in signaling transcriptional control of the expression of key genes, particularly those encoding the unique components of the glycine decarboxylase complex (*GCV1*, *GCV2*, and *GCV3*)[11]. It is not understood how metabolites flow through the various one-carbon metabolic pathways in the cell, but less is known on mechanisms controlling this flow. However, vitamin B12 achieves this regulatory control probably by altering the balance of one-carbon metabolism between the cytoplasm and the mitochondrion to ensure a constant supply of one-carbon units to the important biosynthetic pathways such as those concerned with purine and pyrimidine biosynthesis. It is most likely that mycotoxins may have inhibitory role in these processes.

## Source of Coenzymes in One-Carbon Metabolism

Vitamin $B_{12}$ is a coenzyme for two physiologically important functions in humans: (1) the synthesis of methionine, which is a required step in the production of active folate coenzyme and (2) the conversion of methylmalonic acid to succinic acid. $B_{12}$ assists in transferring the methyl group onto homocysteine to form methionine, which is necessary for the synthesis of myelin sheaths. Vitamin $B_{12}$ is required in the methionine synthase reaction in which homocysteine is converted to methionine and methyl tetrahydrofolate (methyl THF) to THF. The “methyl-folate trap” hypothesis[20,21,22,23] suggests that failure of demethylation of methyl THF with consequent deficiency of folate coenzymes derived from THF is the crucial lesion caused by vitamin $B_{12}$ deficiency. Vitamin $B_{12}$, therefore, serves as a cofactor in the transfer of methyl groups — takes the methyl group from methyl tetrahydrofolate so that THF, the active form of folate, can be used for synthesis of DNA, involved in carbohydrate metabolism. Since vitamin $B_{12}$ is one of the sources of coenzymes, which participate in one-carbon metabolism, then the interaction with mycotoxins probably affects the coenzymatic action[13]. A more recent theory suggests that reduced supply of methionine leads to reduced availability of “activated formate” and hence of formyl THF and it is this defect that results in failure of folate coenzyme synthesis. It is in the supply of THF and not of "active formate" or formyl THF that vitamin $B_{12}$ plays a critical role in folate metabolism[24,25]. It is possible that a substantial majority of the cases of high homocysteine in the older population can be attributed to vitamin $B_{12}$ status[12]. Since the metabolism of a substrate is closely linked to that of its cofactor(s), in the case of homocysteine, the vitamins $B_{12}$ is involved in its metabolism, acting as coenzymes[26]. Since homocysteine is an intermediate compound formed during the metabolism of methionine, deficiency of vitamin $B_{12}$ is one of the major causes of homocysteine elevation in the elderly population[14]. Conversely, the elderly population is susceptible to mycotoxicity.

## Intracellular Recycling of Methionine

The metabolism and intracellular recycling of methionine requires vitamin $B_{12}$ as cofactor and methyltetrahydrofolate as coreactant[27,28,29]. In fact, both vitamin $B_{12}$ and folic acid were required for the biosynthesis of methionine from homocysteine[30]. The beneficial effects of folate in the above conditions can be explained largely within the context of folate-dependent pathways, such as methionine, purine, and pyrimidine biosynthesis[31]. However, the precise detail of folate metabolism is extremely complex and difficult to study because folate-dependent one-carbon metabolism is compartmentalized, involves an enormous number of low-abundance, difficult-to-measure, highly labile folyl coenzymes, and is the subject of genetic variability[31].

## Vitamin $B_{12}$ in Methylation

Tetrahydrofolate (folic acid derivatives) in conjunction with vitamin $B_{12}$ are involved in the methylation of homocysteine to methionine. Since this reaction is $B_{12}$ dependent[27,28] and since mycotoxins interfere with vitamin $B_{12}$ function (by implication and derivation), there is no doubt that these important biochemical reactions would be affected adversely. Deficiency of vitamin $B_{12}$ causes serious and often irreversible neurological disorders, such as burning pain or loss of sensation in the extremities, weakness, spasticity and paralysis, confusion, disorientation, and dementia[3]. Vitamin $B_{12}$ is not only required both in the methylation of homocysteine to methionine, but also in the synthesis of S-adenosylmethionine. Furthermore, since homocysteine is a sulfhydryl-containing amino acid that is formed by the demethylation of methionine, it is normally catalyzed to cystathionine by cystathionine beta-synthase, a pyridoxal phosphate-dependent enzyme, and it is also remethylated to methionine by methionine synthase, a vitamin $B_{12}$–dependent enzyme and by methylenetetrahydrofolate reductase[32]. Vitamin $B_{12}$ deficiency and genetic defects such as cystathionine beta-synthase or abnormality of methylene-tetrahydrofolate reductase or some vitamin $B_{12}$ metabolism defects may contribute to increasing plasma homocysteine levels[32]. S-adenosylmethionine is involved in numerous methylation reactions involving proteins, phospholipids, DNA, and neurotransmitter metabolism. Both vitamin $B_{12}$ and folate deficiency may cause similar neurological and psychiatric disturbances, including depression, dementia, and a demyelinating myelopathy. A current theory[33] proposes that a defect in methylation processes is central to the biochemical basis of the neuropsychiatry of these vitamin deficiencies. Vitamin $B_{12}$ deficiency may specifically affect central monoamine metabolism and aggravate depressive disorders. In addition, the neurotoxic effects of homocysteine may also play a role in the neurological and psychiatric disturbances that are associated with vitamin $B_{12}$ deficiency[33,34,35].

## Vitamin $B_{12}$ in the Prevention of Chromosome Breakage

Folic acid plays a critical role in the prevention of chromosome breakage and hypomethylation of DNA. This activity is compromised when vitamin $B_{12}$ concentration is low because methionine synthase activity is reduced, lowering the concentration of S-adenosylmethionine, which in turn may diminish DNA methylation and cause folate to become unavailable for the conversion[36]. The most plausible explanation for the chromosome-breaking effect of low folate is excessive uracil misincorporation into DNA, a mutagenic lesion that leads to strand breaks in DNA during repair. Both *in vitro* and *in vivo* studies with human cells clearly show that folate deficiency causes expression of chromosomal fragile sites, chromosome breaks, and excessive uracil in DNA, micronucleus formation, and DNA hypomethylation[36]. Deficient levels of folic acid and vitamin $B_{12}$ are associated with elevated chromosome damage rate and high concentrations of homocysteine in the blood. Therefore, elevated homocysteine status, in the absence of vitamin deficiency and low, but not deficient, vitamin $B_{12}$ status are important risk factors for increased chromosome damage in lymphocytes[37].

## CONCLUSION

The roles played by vitamin $B_{12}$ in one-carbon metabolism have been discussed. The overall functions of vitamin $B_{12}$ as a source of coenzymes, in intracellular recycling of methionine, in methionine synthase reaction, in the prevention of chromosome breakage, in methylation, and in maintaining a one-carbon metabolic balance have been reviewed. Although, these roles are evidently documented, the effects of mycotoxins on them are yet to be fully realized in the light of ongoing research. However, the possible adverse interruption of these metabolic roles by mycotoxins have been assessed and evaluated. By implication and derivations from the literature, it is likely that the interruption of the structure and function of vitamin $B_{12}$ would in turn interfere with the one-carbon metabolism leading to the neurological manifestations.

## BIOSKETCHES

**Ebere C. Anyanwu, PhD,** is a Clinical Neurophysiologist and Director of Cahers Neurosciences Research Inc, Houston. He is also an Adjunct Professor of Anatomy and Physiology at the North Harris and Montgomery Colleges in Houston and The Woodlands, respectively. Also a Researcher at the Biomarkers Research Laboratory, Department of Environmental Chemistry and Toxicology, Texas Southern University, Houston. E-mail:  is a Clinical Neurophysiologist and Director of Cahers Neurosciences Research, Inc., Conroe, TX. He is also an Adjunct Professor of Anatomy and Physiology at the North Harris and Montgomery Colleges in Houston and The Woodlands, respectively. E-mail: ebereanyanwu@msn.com

**Mohammed Morad, MD** is a specialist in family medicine, lecturer in family medicine at the National Institute of Child Health and Human Development; Division of Community Health, Ben Gurion University of the Negev and the medical director of a large area clinic in the city of Beer-Sheva. Publications include those on Bedouin health, health aspects, spiritual health and aging in persons with intellectual disability, and he is a presenter on topics like health policy and services for disadvantaged at national and international conferences. E-mail: Tmorad62@barak-online.net

**Andrew W. Campbell**, **MD,** is the Chief Executive and Medical Director of the Medical Center for Immune and Toxic Disorders, Spring, TX. Hs is also a specialist in neuroimmunotoxicology.
